# Cardiomyocyte renewal in the failing heart: lessons from the neonate?

**DOI:** 10.1007/s12551-020-00739-9

**Published:** 2020-07-17

**Authors:** Wouter Derks, Francesca Murganti, Olaf Bergmann

**Affiliations:** 1 Center for Regenerative Therapies Dresden, TU-Dresden, Fetscherstrasse 105, 01307 Dresden, Germany; 2 Karolinska Institutet, Biomedicum, Cell and Molecular Biology, SE-17177 Stockholm, Sweden

The renewal capacity of the heart is extremely limited, with less than 1% cardiomyocyte turnover per year in an adult human heart (Bergmann et al. 2015). This process is thought to be mediated by the proliferation of existing cardiomyocytes rather than by differentiating stem cells (Eschenhagen et al. 2017; Senyo et al. 2013).

One of the hallmarks of heart failure is the loss of functional cardiomyocytes. Whether cardiac disease supports cardiomyocyte renewal is a matter of ongoing debate (Eschenhagen et al. 2017); however, many studies have reported increased cell cycle activity after cardiac injury (Senyo et al. 2013; Vujic et al. 2018).

Intriguingly, a failing heart shows some resemblance to a neonatal heart (Table 1). As observed in the fetal heart (Sturzu et al. 2015), neonatal cardiomyocytes can still duplicate, thereby regenerating the injured heart (Porrello et al. 2011, 2013). During postnatal maturation, cardiomyocytes undergo marked changes in metabolism, redox state, sarcomeric protein expression, and morphology (Guo and Pu 2020; Karbassi et al. 2020). Throughout this process, cardiomyocytes gradually lose their proliferative capacity (Alkass et al. 2015; Karbassi et al. 2020). In failing hearts, certain conditions, including ischemia, hypertrophy, and atrophy, can induce the reactivation of fetal gene programs, leading to partial reversal of morphological differentiation and metabolic maturation processes (Kubin et al. 2011; Taegtmeyer et al. 2010).

**Table 1:** Myocardial characteristics of the neonatal, adult, and failing heart

	Neonatal heart	Adult heart	Failing heart
Extracellular matrix	- Soft - Low number of fibroblasts - Neonatal fibroblast subtype	- Stiff - High number of fibroblasts - Adult fibroblast subtype	- Extensive remodeling of ECM - Increased number of fibroblasts - Activated myofibroblasts
Metabolism	- Glycolysis - Low number of mitochondria	- Oxidative phosphorylation - High number of mitochondria	- Switch towards glycolysis - Mitochondrial dysfunction
Cardiomyocyte morphology	- No T-tubules - Low length-to-width ratio	- Rod shape - T-Tubules - High length-to-width ratio - Increase in cardiomyocyte size	- Further enlargement of cardiomyocyte - Loss of sarcomere organization - Cardiomyocyte structural degeneration
Contractility	- Low contractility - Low β-MHC to α-MHC ratio†	- High contractility - High β-MHC to α-MHC ratio† - ssTnI to cTnI switch	- Reduced contractility - Decreased β-MHC to α-MHC ratio†
Cell cycle	- Cell cycle activity - Proliferation of cardiomyocytes - Mostly mononucleated diploid cardiomyocytes	- Very low cell cycle activity and cardiomyocyte proliferation - Mostly multinucleated and polyploid cardiomyocytes	- Increased cell cycle activity (relative to non-diseased adult hearts) - Some evidence for increased cardiomyocyte proliferation - Increase in nuclear ploidy

^†^Human-specific isoform switch

Nevertheless, we know that increased cell cycle activity in diseased and injured hearts does not lead to efficient regeneration, most likely because cell cycle activity mainly leads to polyploidy and not to substantial proliferation of existing cardiomyocytes (Herget et al. 1997; Hesse et al. 2012; Meckert et al. 2005) (Table 1). However, recent work suggests that mononuclear diploid cardiomyocytes, which are mainly present in early neonatal hearts, retain their capacity to divide (Bersell et al. 2009; Kuhn et al. 2007; Patterson et al. 2017). Based on these findings, the “subpopulation” theory emerged, stating that mononucleated diploid cardiomyocytes are a reservoir to generate new cardiomyocytes (Gan et al. 2019; Patterson and Swift 2019). If this theory was true, only a small fraction of cardiomyocytes would be able to divide in the diseased heart, making it difficult to generate sufficient numbers of new cardiomyocytes to support heart regeneration.

Mature adult cardiomyocytes are rod-shaped, with a high length-to-width ratio, develop invaginations in the form of T-tubules, and are densely packed with myofibrils and mitochondria (Karbassi et al. 2020) (Table 1). However, successful mitosis and cytokinesis require extensive morphological changes, including the disassembly of sarcomeres, to allow symmetric assembly of the mitotic spindle and formation of the contractile ring (Ahuja et al. 2004; Green et al. 2012). Thus, without substantial remodeling, successful cytokinesis seems to be incompatible with the size and shape of adult cardiomyocytes. Importantly, loss of sarcomere organization and disrupted ultrastructure of cardiomyocytes have been documented in patients with failing hearts (Hein et al. 2009), suggesting that one requisite for cardiomyocyte division is present in the diseased heart. A recent study by Gabisonia et al. showed that dedifferentiation of mature cardiomyocytes can be triggered by the delivery of microRNA-199a in large mammals, leading to reentry into the cell cycle. However, these cells resembled a pool of poorly differentiated cardiomyocytes with a myoblastic phenotype, which results in fatal arrhythmias (Gabisonia et al. 2019), demonstrating that a reversal of cardiomyocyte maturation needs to be temporally and spatially tightly controlled to efficiently regenerate the heart.

In concert with the cellular changes in the myocyte, the composition of the extracellular matrix (ECM) is an important factor in regulating cardiomyocyte maturation and cardiomyocyte cell cycle activity (Bassat et al. 2017; Dixon et al. 2019; Morikawa et al. 2017; Yahalom-Ronen et al. 2015). Several components of the neonatal ECM, such as agrin (Bassat et al. 2017) and periostin (Kuhn et al. 2007), have been implicated in augmented cardiomyocyte cell cycle activity. Remodeling of the ECM in cardiac pathologies is controlled by cardiac fibroblasts, and a recent study revealed that a switch in fibroblast subtype from a neonatal to adult state controls cardiomyocyte maturation and cell cycle activity (Wang et al. 2020). ECM synthetized by postnatal cardiac fibroblasts inhibited cardiomyocyte proliferation and increased cardiomyocyte binucleation, indicating that during postnatal maturation the ECM becomes a non-permissive environment for mitotic rounding and cytokinesis of cardiomyocytes (Wu et al. 2020). As the phenotype and number of fibroblasts fundamentally change during aging and in heart disease (Bergmann et al. 2015; Kanisicak et al. 2016), it will be important to investigate how the ECM composition and crosstalk between fibroblasts and cardiomyocytes can be manipulated to devise new regenerative strategies for diseased hearts.

There are some unexpected similarities between regenerative neonatal hearts and adult failing hearts. However, only limited regeneration is observed in the failing heart. It is evident that cardiac regeneration does not depend solely on one factor, but rather on the interplay of changes occurring in the cardiomyocyte and its microenvironment. Future research needs to show how to orchestrate these factors to not only mimic distinct neonatal features of regeneration (e. g., metabolic switch, disassembly of sarcomeric organization) but also achieve complete regeneration of an adult heart.

## References

[CR1] Ahuja P, Perriard E, Perriard JC, Ehler E (2004). Sequential myofibrillar breakdown accompanies mitotic division of mammalian cardiomyocytes. J Cell Sci.

[CR2] Alkass K, Panula J, Westman M, Wu TD, Guerquin-Kern JL, Bergmann O (2015). No evidence for cardiomyocyte number expansion in preadolescent mice. Cell.

[CR3] Bassat E (2017). The extracellular matrix protein agrin promotes heart regeneration in mice. Nature.

[CR4] Bergmann O (2015). Dynamics of cell generation and turnover in the human heart. Cell.

[CR5] Bersell K, Arab S, Haring B, Kuhn B (2009). Neuregulin1/ErbB4 signaling induces cardiomyocyte proliferation and repair of heart injury. Cell.

[CR6] Dixon IMC, Landry NM, Rattan SG (2019). Periostin reexpression in heart disease contributes to cardiac interstitial remodeling by supporting the cardiac myofibroblast phenotype. Adv Exp Med Biol.

[CR7] Eschenhagen T (2017). Cardiomyocyte regeneration: a consensus statement. Circulation.

[CR8] Gabisonia K et al (2019) MicroRNA therapy stimulates uncontrolled cardiac repair after myocardial infarction in pigs. Nature. 10.1038/s41586-019-1191-6 10.1038/s41586-019-1191-6 PMC6768803 31068698

[CR9] Gan P, Patterson M, Sucov HM (2019) Cardiomyocyte polyploidy and implications for heart regeneration. Annu Rev Physiol. 10.1146/annurev-physiol-021119-034618 10.1146/annurev-physiol-021119-034618 31585517

[CR10] Green RA, Paluch E, Oegema K (2012). Cytokinesis in animal cells. Annu Rev Cell Dev Biol.

[CR11] Guo Y, Pu WT (2020). Cardiomyocyte maturation: new phase in development. Circ Res.

[CR12] Hein S (2009). Deposition of nonsarcomeric alpha-actinin in cardiomyocytes from patients with dilated cardiomyopathy or chronic pressure overload. Exp Clin Cardiol.

[CR13] Herget GW, Neuburger M, Plagwitz R, Adler CP (1997). DNA content, ploidy level and number of nuclei in the human heart after myocardial infarction. Cardiovasc Res.

[CR14] Hesse M (2012). Direct visualization of cell division using high-resolution imaging of M-phase of the cell cycle. Nat Commun.

[CR15] Kanisicak O (2016). Genetic lineage tracing defines myofibroblast origin and function in the injured heart. Nat Commun.

[CR16] Karbassi E, Fenix A, Marchiano S, Muraoka N, Nakamura K, Yang X, Murry CE (2020). Cardiomyocyte maturation: advances in knowledge and implications for regenerative medicine. Nat Rev Cardiol.

[CR17] Kubin T (2011). Oncostatin M is a major mediator of cardiomyocyte dedifferentiation and remodeling. Cell Stem Cell.

[CR18] Kuhn B, del Monte F, Hajjar RJ, Chang YS, Lebeche D, Arab S, Keating MT (2007). Periostin induces proliferation of differentiated cardiomyocytes and promotes cardiac repair. Nat Med.

[CR19] Meckert PC, Rivello HG, Vigliano C, Gonzalez P, Favaloro R, Laguens R (2005). Endomitosis and polyploidization of myocardial cells in the periphery of human acute myocardial infarction. Cardiovasc Res.

[CR20] Morikawa Y, Heallen T, Leach J, Xiao Y, Martin JF (2017). Dystrophin-glycoprotein complex sequesters Yap to inhibit cardiomyocyte proliferation. Nature.

[CR21] Patterson M, Swift SK (2019). Residual diploidy in polyploid tissues: a cellular state with enhanced proliferative capacity for tissue regeneration?. Stem Cells Dev.

[CR22] Patterson M (2017). Frequency of mononuclear diploid cardiomyocytes underlies natural variation in heart regeneration. Nat Genet.

[CR23] Porrello ER, Mahmoud AI, Simpson E, Hill JA, Richardson JA, Olson EN, Sadek HA (2011). Transient regenerative potential of the neonatal mouse heart. Science.

[CR24] Porrello ER (2013). Regulation of neonatal and adult mammalian heart regeneration by the miR-15 family. Proc Natl Acad Sci U S A.

[CR25] Senyo SE et al (2013) Mammalian heart renewal by pre-existing cardiomyocytes. Nature. 10.1038/nature11682 10.1038/nature11682 PMC3548046 23222518

[CR26] Sturzu AC (2015). Fetal mammalian heart generates a robust compensatory response to cell loss. Circulation.

[CR27] Taegtmeyer H, Sen S, Vela D (2010). Return to the fetal gene program: a suggested metabolic link to gene expression in the heart. Ann N Y Acad Sci.

[CR28] Vujic A (2018). Exercise induces new cardiomyocyte generation in the adult mammalian heart. Nat Commun.

[CR29] Wang Y (2020). Single-cell analysis of murine fibroblasts identifies neonatal to adult switching that regulates cardiomyocyte maturation. Nat Commun.

[CR30] Wu CC, Jeratsch S, Graumann J, Stainier D (2020) Modulation of mammalian cardiomyocyte cytokinesis by the extracellular matrix. Circ Res. 10.1161/CIRCRESAHA.119.316303 10.1161/CIRCRESAHA.119.316303 32564729

[CR31] Yahalom-Ronen Y, Rajchman D, Sarig R, Geiger B, Tzahor E (2015) Reduced matrix rigidity promotes neonatal cardiomyocyte dedifferentiation, proliferation and clonal expansion. Elife 4. 10.7554/eLife.07455 10.7554/eLife.07455 PMC4558647 26267307

